# Osteoadherin Accumulates in the Predentin towards the Mineralization Front in the Developing Tooth

**DOI:** 10.1371/journal.pone.0031525

**Published:** 2012-02-15

**Authors:** Hero Nikdin, Marie-Louise Olsson, Kjell Hultenby, Rachael V. Sugars

**Affiliations:** 1 Oral Biology, Department of Dental Medicine, Karolinska Institutet, Huddinge, Sweden; 2 Department of Laboratory Medicine, Clinical Research Centre, Karolinska Institutet, Huddinge University Hospital, Stockholm, Sweden; University of South Florida College of Medicine, United States of America

## Abstract

**Background:**

Proteoglycans (PG) are known to be involved in the organization and assembly of the extracellular matrix (ECM) prior to mineral deposition. Osteoadherin (OSAD), a keratan sulphate PG is a member of the small leucine-rich (SLRP) family of PGs and unlike other SLRPs, OSAD expression is restricted to mineralized tissues. It is proposed to have a high affinity for hydroxyapatite and has been shown to be expressed by mature osteoblasts but its exact role remains to be elucidated.

**Methodology/Principal Findings:**

We investigated the protein distribution of OSAD in the developing mouse tooth using immunohistochemistry and compared its expression with other SLRPs, biglycan (BGN), decorin (DCN) and fibromodulin (FMD). OSAD was found to be specifically localized in the predentin layer of the tooth and focused at the mineralization front. These studies were confirmed at the ultrastructural level using electron microscopy (iEM), where the distribution of immunogold labeled OSAD particles were quantified and significant amounts were found in the predentin, forming a gradient towards the mineralization front. In addition, iEM results revealed OSAD to lie in close association with collagen fibers, further suggesting an important role for OSAD in the organization of the ECM. The expression profile of mineralization-related SLRP genes by rat dental pulp cells exposed to mineralization inducing factors, showed an increase in all SLRP genes. Indeed, OSAD expression was significantly increased during the mineralization process, specifically following, matrix maturation, and finally mineral deposition. Alizarin Red S staining for calcium deposition showed clear bone-like nodules, which support matrix maturation and mineralization.

**Conclusions:**

These studies provide new evidence for the role of OSAD in the mineralization process and its specific localization in the predentin layer accumulating at the mineralization front highlighting its role in tooth development.

## Introduction

The extracellular matrix (ECM) of skeletal and dental tissues is primarily comprised of a three dimensional network of collagen fibers, where type I collagen (90%) predominates, and the remainder are non-collagenous proteins (NCPs). This highly organized organic matrix becomes encased within the inorganic mineral, hydroxyapatite during the mineralization process [1], [2]. Dentinogenesis is highly controlled by the expression of ECM proteins, which undergo transformations and modifications from the non-mineralized predentin to the mineralized dentin. They also play a role in structural and metabolic functions of mineralized tissues [3], [4]. A number of NCPs, in particular proteoglycans (PGs), have been identified in the predentin and dentin of teeth, where several of them belong to the family of small leucine-rich proteoglycans (SLRPs) [3], [5]. SLRPs have been described multiple functions within mineralized tissues, including cell regulation, matrix organisation, mineral deposition and crystal growth [6]. Within the predentin most PGs are found in irregular patterns, filling the spaces between collagen fibres, compared to PGs in dentin where they are found in close contact with collagen fibres [1].

SLRPs belong to an increasing gene class (17 genes) [7], and each possesses a distinctive leucine-rich structural motif repeat flanked by cysteine residues at the N- and C-terminals. The leucine-rich region consists of 6–10 repeats of 20–29 amino acids and participates in a wide range of biological functions, such as binding with the collagen triple helix [4], [7], [8]. SLRPs also carry one or more glycosaminoglycan (GAG) chains, covalently attached to the core protein, which can influence the PG function depending upon the nature of the GAG chain and molecular localization. SLRPs are recognized as active components of the ECM and are found ubiquitously distributed in all soft and hard mineralized tissues [9], [10]. In addition, SLRPs have been extensively implicated in the biomineralization of dentin; specifically, biglycan (BGN), decorin (DCN), fibromodulin (FMD) and lumican, which have been shown to be involved in dentinogenesis. In particular, SLRPs have been shown to influence collagen fibrillogenesis during ECM formation and organization and to act as regulators of hydroxyapatite binding and mineral crystal growth [3]. In depth studies have shown SLRPs and GAG chains to be differently distributed across the mineralizing tooth from the odontoblast cell layer to predentin, predentin/dentin interface and dentin [3], [10], [11], [12]. Chondroitin sulphate (CS) and dermatan sulphate (DS) GAG chains were found proximal to the predentin, close to the odontoblast cell layer [3]. In comparison, keratan sulphate (KS), the GAG associated with the class II family of SLRPs, produced an increasing gradient towards the mineralization front close to the predentin layer [13], [14].

Osteoadherin (OSAD), a 47 kDa KS-SLRP, was identified and purified from bovine long bones by Wendel et al. [15] and was found specifically located to mineralized tissues [15], [16]. The function of OSAD during mineralization remains to be fully elucidated; however it has been shown to have a high affinity for hydroxyapatite, via the large and acidic C-terminal [15], [16]. OSAD mRNA expression has been detected within mature osteoblasts [15], [16], [17] and in similarity to other SLRPs, OSAD was postulated to bind to the triple helical collagen fibres and stabilize the overall architecture of the ECM, although this remains to be determined. The presence of OSAD in the mineralized bone matrix is similar to that of bone sialoprotein (BSP) [18]. BSP has been shown to be a marker for mature osteoblasts [19], [20], hence the close association with OSAD further strengthens its role as a mineralization specific marker.

Studies have explored the localisation of OSAD in the tooth and it has been shown to be expressed in mature human odontoblasts and also present during the development of rat molars [21], [22], [23]. However, the exact role and distribution of OSAD during complete tooth formation has yet to be fully determined, nevertheless mRNA expression of OSAD has been shown in mature rat odontoblasts responsible for the secretion and mineralization of dentin. In addition, an increasing gradient of OSAD mRNA was also observed as the cells matured, showing the highest expression in the secretory odontoblasts synthesizing the predentin [21]. In support of the gene expression studies, OSAD protein localization showed the same distribution pattern, where OSAD was present in the alveolar bone surrounding the molar from a 19-day-old rat embryo [23], and an increasing gradient was found together with odontoblast maturation in the rat [23].

Despite these insights, studies on OSAD distribution during development are limited and light and electron immunohistochemistry provide useful tools for visualizing SLRPs and to study them further. This study aimed to clarify the localization of OSAD in mouse within the developing tooth from the early cap stage, and to compare the distribution with mineralization-associated SLPRs with a view to provide further evidence for the role of OSAD during dentinogenesis. Furthermore, in an attempt to elucidate the putative role for OSAD in mineralization, we examined its expression within an *in vitro* mineralizing pulp cell culture.

## Results

### OSAD is confined to the predentin ECM of the tooth and is identified with the onset of dentinogenesis

**Figure 1:**
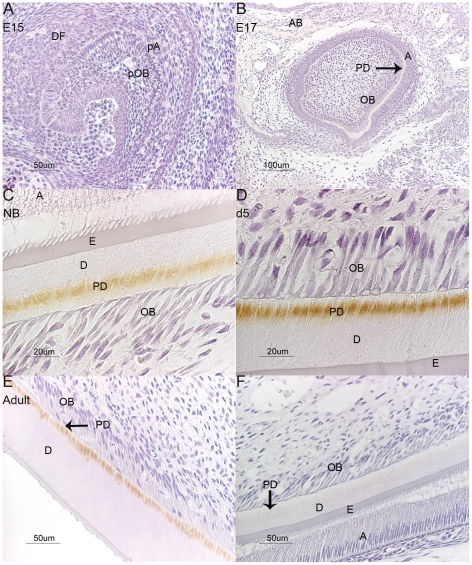
Immunostaining of OSAD in the developing mouse tooth. No signal was detected for OSAD at the early bell stage of the tooth E15 (A), OSAD was first localized weakly within the alveolar bone at E17 (B), and once dentinogenesis was initiated, it continued throughout crown formation from NB (C), d5 (D) and into the adult (F). The expression of OSAD was restricted to the predentin layer in all developmental phases, Control (F) sections were incubated with OSAD antibody (0.2 µg/µl) in the presence of 10× excess of recombinant mouse OSAD protein (0.1 µg/µl). A = ameloblasts, AB = alveolar bone, D = dentin, DF = dental follicle, E = enamel, pA = pre-ameloblasts, PD = predentin, pOB = pre-odontoblasts and OB = odontoblasts.

Mouse mandibles were collected at embryonic (E) days E15, E17, newborn (NB), day 5 (d5) and adult (>3months), and immunohistochemistry localization studies with an antibody targeted against mouse OSAD were performed at all these time points (Fig. 1). The specificity of the antibody was demonstrated by Western blot analysis of protein extracts from mouse incisors and against the recombinant protein used to raise the antibody (Fig. S1). OSAD was detected in the organic fraction of the tooth (predentin (GuHCl) extraction) and revealed a weak band at 80 kDa, whereas protein extracts that were mineral bound, including the predentin/dentin interface and dentin (EDTA extraction) exhibited a more intense band also at 80 kDa. This supports the notion that OSAD exits as a glycosylated protein, with KS GAG chains and N–linked glycosylation within mineralized tissues [16]. The highly glycosylated nature of OSAD was further observed in the recombinant mouse protein that showed bands at approximately, 120 kDa, 80 kDa, 60 kDa and 46 kDa corresponding to the bands expected for KS-substitution, N-linked glycosylation and the core protein respectively. Digestions with N-glycosidase and keratinase for N-linked glycosylation and KS-GAG chains confirmed these post-translational modifications (data not shown).

OSAD was first detected by immunohistochemistry, weakly within the alveolar bone of E17 mice surrounding the developing tooth, and this expression continued throughout the rest of the stages examined (Fig. 1). OSAD was not localized in the tooth until dentinogenesis was well-established and crown formation was progressing in NB mice. Here the protein was localized to the predentin phase, distal to the odontoblast cell body, with little or no staining apparent in the cells. With crown maturation (d5 and adult) OSAD localization continued to be focused within the predentin and particularly defined to the distal predentin/dentin interface. Data is shown for the incisors only, however we also examined molar development and it was apparent that the initial expression of OSAD was observed in NB mice, and this was equivalent to the late bell stage early dentinogenesis. The protein was again localized within the predentin and this continued with dentinogenesis and ameleogenesis in d5 and into adult mice.

**Figure 2:**
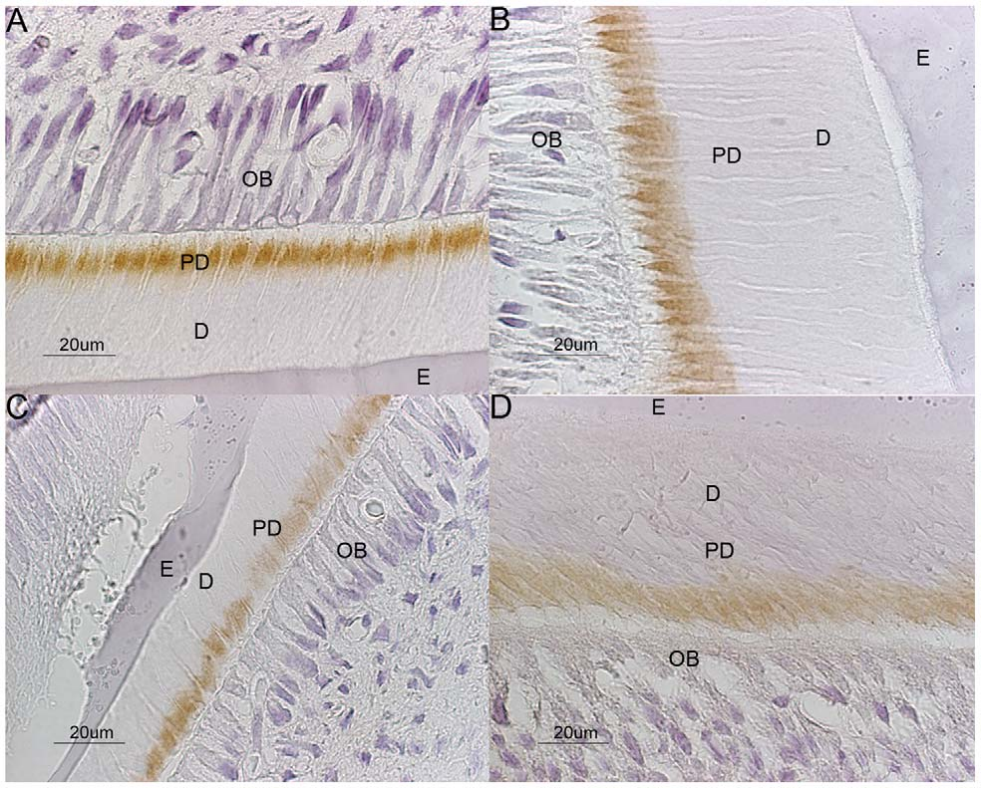
Immunostaining of OSAD in comparison to the other SLRPs in d5 mice. OSAD (A) expression is clearly noted in the predentin towards the predentin/dentin interface, BGN (B), DCN (C) and FMD (D) were detected again in the predentin but more closely located to the odontoblastic cell layer. A = ameloblasts, AB = alveolar bone, D = dentin, DF = dental follicle, E = enamel, pA = pre-ameloblasts, PD = predentin, pOB = pre-odontoblasts and OB = odontoblasts.

Comparison of OSAD expression throughout development with the other SLRPs, BGN, DCN and FMD was performed (Fig. 2, Figs. S2, S3, S4 and Table 1). These antibodies were kindly provided by Larry Fisher (NIDCR, NIH) and their specificity has been previously defined [24]. In similarity to OSAD, BGN was not detected at the early stages of development (E15 and E17) but once crown formation was initiated (Fig. S2). BGN was mostly restricted to the predentin but proximal to the odontoblast cell layer throughout development and into the late crown stage. Alveolar bone was weakly stained from E17, which increased with the later stages of development. BGN localization during molar development followed the profile observed for the incisors. However, an intensified prominent signal was detected by the adult stage, and BGN was also faintly seen in the pulp chamber and surrounding alveolar bone (data not shown). In comparison, DCN was observed at an earlier developmental stage of E15 (Fig. S3). At this time point, staining for DCN was apparent in the surrounding cells of the dental follicle and by E17, at the late bell stage, with early dentinogenesis, positive labeling was present in the pulp complex and in the first secreted predentin. By E17 in the molars, corresponding to the bud to cap transition stage, DCN was observed in the surrounding alveolar bone. With early dentinogenesis, in both the incisors and molars, DCN was found localized to the odontoblastic cell layer and within the predentin. As the tooth matured, DCN staining was observed throughout the predentin layer and also the surrounding alveolar bone (d5). The distribution of FMD throughout all developmental stages was similar to BGN. Positive signal was not seen until the NB stage (Fig. S4). Here FMD was detected in the pulp and alveolar bone as well as predentin and dentin. A similar pattern was seen in the molars, where FMD was restricted to the predentin and proximal borders of the odontoblastic cell layer. With tooth maturation in adult mice, FMD was observed in the pulp chamber in the molars and the surrounding alveolar bone.

**Table 1:** Semi-quantitative observations comparing immunohistochemistry staining in developing mouse incisors.

	DP/Dental pulp	pOB/OB	PD	Dentin	Enamelorgan	pA/A	Enamel	DF/AB
**E15**								
OSAD	−	−	N/A	N/A	−	−	N/A	−
BGN	−	−	N/A	N/A	−	−	N/A	−
DCN	−	−	N/A	N/A	−	+	N/A	±
FMD	−	−	N/A	N/A	−	−	N/A	−
**E17**								
OSAD	−	−	−	N/A	−	−	N/A	±
BGN	−	−	−	N/A	−	−	N/A	±
DCN	++	+	++	N/A	+ SI	+	N/A	+
FMD	+	±	−	N/A	± SI	−	N/A	±
**NB**								
OSAD	−	−	+++	+	−	−	−	++
BGN	+	−	+++	++	−	−	−	−
DCN	−	−	+++	±	−	−	−	++
FMD	++	++	+++	++	±	−	−	+
**D5**								
OSAD	−	−	+++	++	−	−	−	+
BGN	−	−	+++	±	−	−	−	−
DCN	−	−	+++	−	±		−	++
FMD	−	−	+++	++	−	−	−	++
**Adult**								
OSAD	−	−	+++	++	N/A	N/A	N/A	−
BGN	−	−	++	±	N/A	N/A	N/A	±
DCN	−	−	+++	−	−	−	−	±
FMD	++	++	+++	++	−	−	−	−

Scoring - very strong (+++), strong (++), weak (+), more or less detectable with some variation (±), absent (−) and not applicable (N/A). A = ameloblasts, AB = alveolar bone, DF = dental follicle, DP = dental papilla, OB = odontoblasts, pA = pre-ameloblasts, pOB = pre-odontoblasts, SI = stratum intermedium.

### Ultrastructural analysis (iEM) revealed that OSAD accumulated at the mineralization front with tooth development

**Figure 3:**
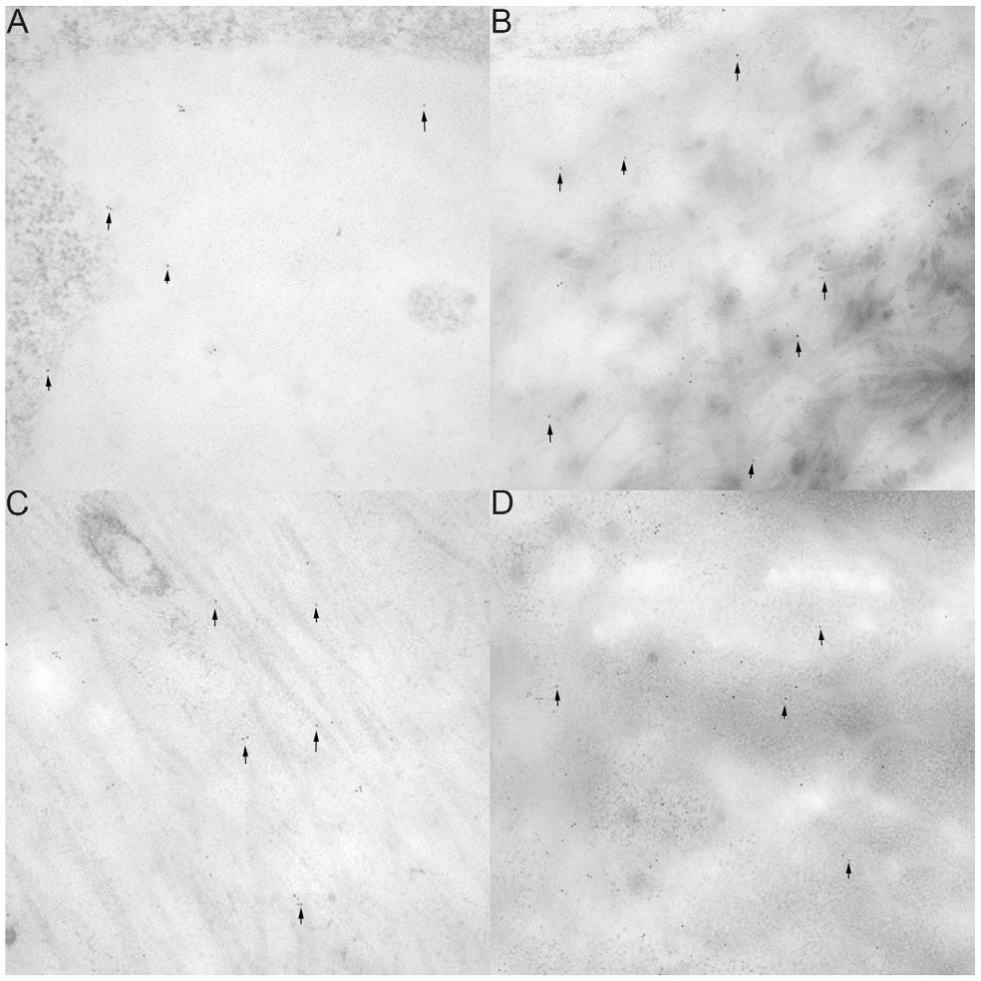
Electron images of OSAD in mouse predentin at different developmental stages. Electron images illustrating gold-labeled OSAD in the highly active predentin throughout all the different developmental stages examined (E15 (A), NB (B), D5 (C) and adult (D)). The number of gold-particles detected increased with development from E15 to NB. The elevated levels of OSAD expression were maintained in the predentin to d5 and adulthood. Localization of OSAD close to collagen fibers are very clearly observed in d5 mouse (C).

**Figure 4:**
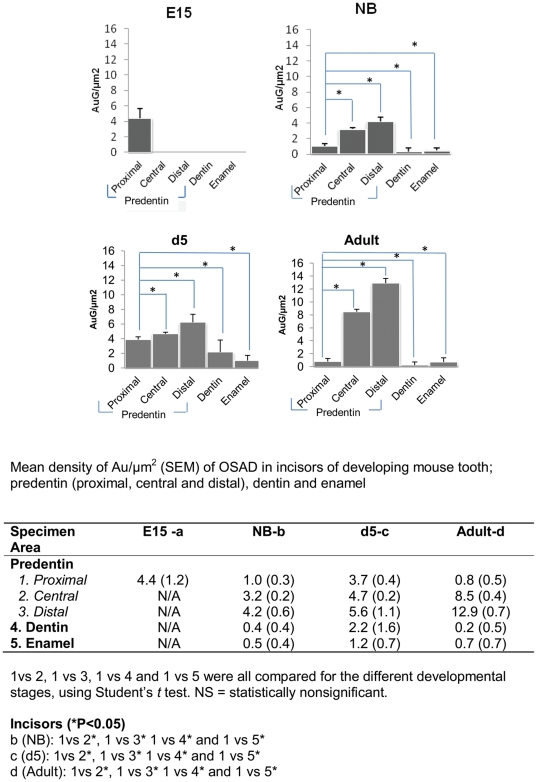
Quantification of OSAD expression in the developing incisor. Quantification of the gold-labeled particles in the predentin (proximal, central and distal), dentin and enamel following ultrastructural analysis of E15, NB, d5 and adult incisors. The results are expressed as number of particles/µm^2^ (Au/µm^2^). Statistically significant differences p<0.05 are denoted by *.

OSAD antibodies were detected by gold conjugated secondary antibodies at all developmental stages of tooth formation, albeit there were considerable differences in the distribution of OSAD across the various tooth compartments (predentin (proximal, central and distal regions), dentin and enamel) (Figs. 3, 4, S5 and S6). Quantification of the gold particles is provided in Fig. 4 for the incisors and in Fig. S6 for the molars. At all developmental stages (E15, NB, d5 and Adult), negligible numbers of gold particles were detected associated with the odontoblast cell body (data not shown). In addition, very few gold particles were detected in the enamel. OSAD was mainly localized to the developing predentin and dentin. During NB tooth development, a gradient of gold particles was clearly evident in the predentin, from the proximal and central regions to the most distal at the mineralization front. This gradient was maintained into adulthood in both the incisor and molar. Interestingly the number of gold particles detected in the dentin was significantly less (P<0.05) than observed in the predentin, which supports the data from the light microscopy studies. Importantly, OSAD was found in close association to collagen fibers in both the predentin and dentin layers, which maintains the notion that OSAD, may be involved in collagen fibrillogenesis, although this remains to be functionally determined. Of note, adsorption controls with the recombinant OSAD protein were performed in parallel but the number of gold labeled particles was negligible (Fig. S5).

### Induction of mineralizing rat dental pulp (rDPC) cultures stimulates SLRP gene expression with matrix maturation and mineral deposition

**Figure 5:**
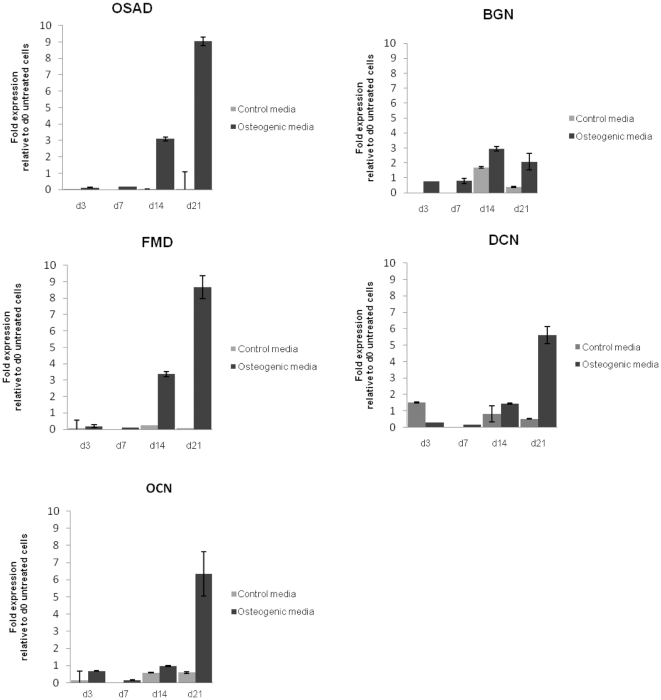
Gene expression of OSAD increased when induced with mineralizing media in rDPC. rDPC were left to differentiate for 21 days in media supplemented with 50 µg/ml ascorbic acid phosphate and 10 mM β-glycerophosphate to induce a mineralizing phenotype. The changes in gene expression were measured after d3, 7, 14 and 21. Data showed an increased expression of all SLRPs (BGN, DCN, FMD and OSAD) over this period, in particular OSAD was highly expressed as the rDPC entered matrix maturation and mineralization. OCN, a mineralized tissue specific marker, also demonstrated a significant increase in expression with the onset of mineralization.

Gene expression was assessed in mineralizing-induced rDPC cultures after 3, 7, 14 and 21 days (Fig. 5). Levels of OSAD, BGN, DCN, FMD and osteocalcin (OCN) gene expression were analyzed to evaluate fold-change as cells entered matrix maturation and mineralization. Data was normalized to GAPDH using d0 as a calibrator, and the data was performed in triplicate. Expression profiles for each gene showed a similar profile across the different time points, even though the fold-change varied. No significant changes in gene expression levels were seen between d3 and d7 in the rDPC cultures. However by d14 and d21, a significant increase in all genes was observed, up to 9-fold (Fig. 5), specifically for OSAD and FMD. Interestingly OSAD and FMD demonstrated a similar increase and expression profile as OCN, a highly specific molecule involved in biomineralization.

**Figure 6:**
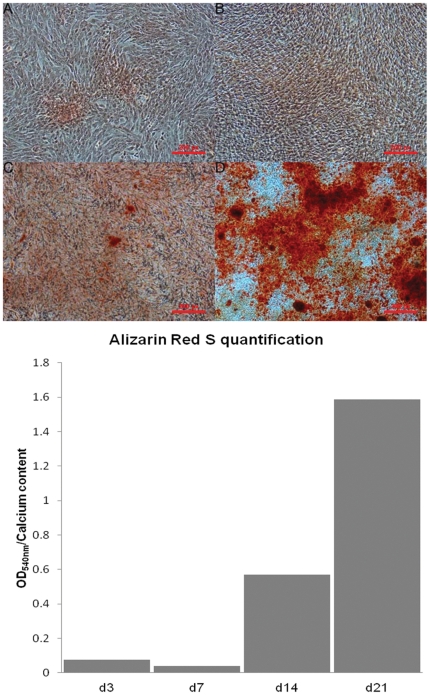
Alizarin Red S staining of rDPC in mineralizing culture. Mineral deposition was examined using Alizarin Red S staining. rDPC were investigated after d3 (A), d7 (B), d14 (C) and d21 (D). Clear mineralizing nodules were observed in the osteogenically induced cultures at d21 (D). Alizarin Red S quantification demonstrated elevated calcium content by nearly 10-fold at d14 and d21 compared to d3 cultures.

Nodule formation and the mineralization capacity of the rDPC were assessed with Alizarin Red S staining (Fig. 6), which stains deposited calcium red. rDPC grown in mineral-inducing media did not show calcium depositions until d14 with matrix maturation, however by d21, clear nodules, indicated mineral deposition compared to rDPC cultures at d3 and d7. Quantification of the staining demonstrated the significant accumulation of calcium (Fig. 6).

## Discussion

Previous studies have implicated OSAD during tooth development and this study was designed to provide additional information as to the role of OSAD during tooth formation and dentinogenesis. In particular we have focused on the quantitative analysis of OSAD distribution by iEM. We also present a comparative analysis of OSAD localization against other SLRP family members principally implicated in dentinogenesis, BGN, DCN and FMD throughout all developmental time points. As a result we present a novel analysis of the localization pattern of these proteins during all stages of tooth formation.

Immunohistochemistry and ultrastructural localization both showed OSAD to be confined within the predentin layer and that the first localization of OSAD was not observed until the secretion of a predentin matrix during early dentinogenesis, this continued throughout crown formation and into the mature erupted tooth. Protein distribution [22], [23] and the gene expression pattern [21], [23] of OSAD have previously been studied in the tooth. Buchaille and colleagues [21] detected OSAD at the mRNA level within polarized and secretory odontoblasts and polarized ameloblasts of developing rat molars from the late bell stage onwards. This study was supported by Petersson and co-workers [22], who also showed OSAD mRNA within secretory odontoblasts but that expression diminished with odontoblast maturation. At the protein level, OSAD was again detected within the cells in a similar pattern to the gene expression, and within the predentin, dentin [22], [23] and enamel [23]. The current study adds to these earlier findings by presenting a detailed overview of OSAD from the earlier cap stage of tooth development. At the dentin secretory stage of tooth formation our data supports those previous studies. However, we were unable to detect OSAD within odontoblasts, and in similarly to Petersson and co-workers [22], in the enamel matrix. The variations witnessed with the previous reports may be attributable to the different antibodies used in the studies. Within the current investigation a high affinity antibody was employed, derived from recombinant mouse OSAD core protein. This antibody was highly specific and characterisation demonstrated that it could detect all glycosylated forms of OSAD, as shown with the Western blot analysis of protein extracts from mouse teeth, which is highly significant since SLRPs are known to be proteolytically processed during dentinogenesis [3], [25].

Comparison of OSAD distribution to other SLRP family members provided an additional insight into the role of these molecules during tooth development. Initiation of OSAD expression coincided with the expression of both BGN and FMD, whereas DCN was expressed much earlier during the late bell stage. While OSAD localization was confined to the predentin, showing the distal accumulation towards the mineralization front, DCN was detected throughout the predentin, and BGN and FMD were proximally located closer to the odontoblast layer. At the light microscope level, few studies have reported the distribution of these SLRPs during tooth development, although their distribution has been extensively examined using iEM. The patterns observed in the current light microscopy study resemble those demonstrated by Goldberg and co-workers [26] in the mouse first molar at post-natal day one. However, their study demonstrated significant levels of BGN, DCN and FMD in the developing enamel organ, which differs from the present study.

Analysis of protein distribution by light microscopy was essential to the current investigation to provide a basis for the ultrastructural study. Indeed it was very apparent from the light microscopy that OSAD formed a gradient across the predentin, accumulating at the predentin/dentin interface. To explore this further we performed in depth quantitative analysis to determine the amount of OSAD present within the major compartments of the tooth by iEM. For the first time we show that indeed a gradient of OSAD localization was present, with the largest number of gold labelled OSAD accumulating within the predentin layer closest to the predentin/dentin interface (distal). Of note, few particles were found in the odontoblast cell itself or within the enamel matrix at all developmental stages, thus supporting our immunohistochemistry results. The gradient distribution of OSAD was extremely interesting considering that other SLRPs, BGN, DCN, lumican and their constituent GAG chains within the mineralized tooth also exhibited a gradient distribution (as reviewed by Embery et al. [3]). DCN was shown to form a gradient distally towards the mineralization front, whereas no such gradient was reported for BGN [3]. However, their constituent C4S/DS GAG chains exhibited a converse gradient distribution, showing greater amounts of the GAG chains closer to the odontoblast cell body [14]. Of the class II SLRPs, antibodies targeted against lumican and the KS-GAG chain revealed an increasing gradient distally [11]. Our results support the above data, as we showed with both light and electron immunolocalization studies that, OSAD was increased near the mineralization front and thus contributes to the pool of KS-GAG chains report by Hall et al. [11].

Ultrastructurally, OSAD was also found in close localization to collagen fibres in the predentin and dentin layers of d5 mice, in support of previous findings [22] and the notion that OSAD may be involved in collagen fibrillogenesis. FMD [27], BGN [28] and DCN [29] have also been shown to be involved in collagen fibrillogenesis, where they bind to different sites on the collagen triple helix fiber [30]. Molecular modeling of SLRPs has demonstrated a curved horseshoe shape, which is proposed to facilitate collagen binding [31]. In particular, DCN has been shown to bind near the C-terminus of type I collagen via leucine rich repeats 4–6 [32], [33], [34]. Whether OSAD binds and influences collagen fibrillogenesis in a similar manner to other SLRPs remains to be functionally determined. To date, no knockout (KO) models for OSAD have been established, although it would be of great interest to see the effects on mineralization in these animal models. Both DCN [35], [36] and FMD [37] KO models, have presented with skin fragility and irregular collagen fibrils, whereas the BGN KO model displayed a reduction in bone mass [38]. Molars from DCN KO mice showed porous dentin, poor mineralization and delayed enamel formation in the developing molars, although the defects recovered by six weeks of age [39], FMD KO also presented with altered dentinogenesis resulting from delayed dentin deposition [37].

Studies have examined the gene expression patterns of OSAD within osteoblastic cells, however to date no studies have examined OSAD within mineralizing dental pulp cells *in vitro*. At the early stages of proliferation and differentiation (d3 and d7) OSAD was expressed at very low levels. This data follows that reported for the mouse calvariae osteoblast cell line (MC3T3-E1), where the over-expression of OSAD suggested a role for the PG in the regulation of cell proliferation and migration [17]. In the dental pulp cells once ECM maturation and mineralization had initiated, the levels of OSAD increased considerably nearly 10-fold by d21. The data for OSAD was similar to that found for OCN, which is considered a specific marker for mature osteoblasts [40]. We can conclude that indeed OSAD can be considered a marker for mineralizing cells. The expression patterns for DCN and FMD were similar to that of OSAD, as they elevated with matrix maturation and mineralization. This is in contrast to the study by Mochida et al. [41], who also used MC3T3-E1 cells but found that DCN significantly decreased during the later stages of mineralization. BGN may have a role in cell proliferation and matrix maturation but to a lesser degree in directing mineral deposition compared to other SRLPs. This finding is consistent with the BGN KO mice that present with a decreased bone mass [42], All these results further supports the essential role of SLRP's in mineralization and how without these full mineralization cannot occur.

The primary role of predentin SLRPs is to facilitate the organization of the ECM collagen matrix and to direct hydroxyapatite binding and crystal growth [3], but the exact role of KS-SLRPs remains unclear. Functional studies investigating OSAD biomolecular interactions have been hindered by the inability to derive the protein in a highly purified state with the expected glycosylation patterns and substitution. Our study has shown, by immunohistochemistry and iEM that the expression of OSAD throughout development is specifically located in the predentin/mineralization front. OSAD is expressed at the early stages of tooth formation and continues to be very specific throughout development and localization. There is also a clear temporal difference between OSAD, DCN, BGN and FMD expression, which suggests defined functions for these SLRPs, however together, these results demonstrate a very specific role for OSAD in biomineralization. Furthermore, we confirm that OSAD is a key marker of mature dental pulp cells *in vitro*. Thus, in the current study we have contributed to the further understanding of this family of molecules by demonstrating that in addition to lumican, OSAD accumulates towards the predentin/dentin interface and may have a very definite role in directing the biomineralization process.

## Materials and Methods

### Animals and Ethics Statement

Tissue samples were collected from the NMR1 strain of mice (Scanbur BK AB, Stockholm, Sweden) at E15, 17, NB, d5 and adult (>3 months). Samples were collected to enable the analysis of tooth and mandible development. The experiments were performed in accordance with the current legislation in Sweden and after approval from the Karolinska Institutet Ethical Research Board.

### Sample preparation for histological analyses

The tissues were fixed in 4% paraformaldehyde for 3–24 h at 4°C depending on the stage of development. The samples were decalcified in 12.5% (w/v) ethylenediaminetetraacetic acid (EDTA) solution, pH 7.0 for 2–14 days, dehydrated and embedded in paraffin. Seven micrometer thin sections were mounted on Super Frost Plus slides (Menzel-Gläser, Braunschweig, Germany). The sections were deparaffinized in xylene and rehydrated through a graded series of alcohols and rinsed in Tris buffered saline (TBS, 50 mM Tris, 150 mM sodium chloride, pH 7.6). Basic histological analysis was performed by staining the sections with Hematoxylin and Eosin.

### Immunohistochemical staining – light microscopy

For immunohistochemical staining, rehydrated sections were pre-treated with 0.2 M HCl for 15 min and rinsed in TBS at room temperature (RT). The antibodies against BGN, DCN and FMD recognize the protein cores which lack GAG chains. These sections were treated with 0.05 U/ml of chondroitinase ABC (from *Proteus vulgaris*; Sigma Aldrich St. Louis, USA), in 50 mM Tris, 60 mM sodium acetate and 0.02% bovine serum albumin, pH 8 for 30 min at 37°C. Sections for all antibodies including OSAD were incubated with 3% H_2_O_2_ for 15 min at RT to eliminate endogenous peroxidise activity.

Non-specific binding was blocked using 4% normal rabbit serum (NRS) (DAKO Glostrup, Denmark) for OSAD and 4% normal goat serum (DAKO) for BGN, DCN and FMD, for 1 h at RT. This was followed by incubation with the primary antibodies against mouse OSAD (1∶300 R&D Systems Inc, Minneapolis, USA, Fig. S1), mouse BGN (1∶1500 LF159) DCN (1∶2000 LF113), and FMD (1∶500 LF150) (all generously provided by Larry Fisher, NIDCR, NIH) [24], prepared in the respective blocking solutions. Control experiments for OSAD were performed with the anti-OSAD antibody (0.2 µg/µl) in the presence of 10× excess of recombinant mouse OSAD (0.1 µg/µl R&D Systems Inc), which was incubated overnight at 4°C. For BGN, DCN and FMD the primary antibodies were omitted. The sections were rinsed three times in TBS for 5 min and subsequently probed with the secondary antibody for 1 h at RT; horseradish-peroxidase (HRP)-conjugated rabbit anti-goat IgG (1∶300, DAKO) for OSAD and HRP-conjugated goat anti-rabbit (1∶300 DAKO) for DCN, BGN and FMD. Bound antibodies were detected using the DAB substrate (DAKO) and sections were counterstained for 10 sec with Mayer's Hematoxylin, and mounted in Pertex® (Histolab Products AB, Gothenburg, Sweden). Photomicrographs were taken with Nikon Eclipse E600 microscope, using Nikon Digital camera DXM 1200. Staining intensity was evaluated as previously reported [37] and scored as very strong (+++), strong (++), weak (+), more or less detectable with some variation (±), absent (−) and not applicable (N/A).

### Ultrastructural localization

Mandibles from the corresponding time points were dissected out and fixed in 3% paraformaldehyde in 0.1 M sodium phosphate buffer (PB), pH 7.4 overnight. The tissues were demineralized for 2–14 days in 12.5% EDTA prior to dehydration in methanol and embedding in Lowicryl K11M (Polysciences, Inc, Warringtown) at low temperature as described by Hultenby et al. [43]. Ultrathin sections were cut and placed on formvar coated one-hole nickel grids. Immunolabeling was performed by first blocking with NRS (DAKO) for 30 min, followed by incubation with goat anti-mouse OSAD antibody 1∶30 in 0.1 M phosphate buffer containing 0.1% NRS (PBR) at 4°C overnight. Grids were washed in PBR and the primary antibody was detected with rabbit anti-goat antibody conjugated with 10 nm gold particles (BB International, England) for 2 h and washed in PB and post-fixed. Absorption control experiments were performed by incubating the primary antibody with an excess of the recombinant mouse OSAD. Sections were examined in a Tecnai 12 (FEI Company, The Netherlands). A pilot study was performed to determine the number of images needed for an appropriate sample using cumulative mean plot for evaluation [44]. Thus, 10 randomly images from each compartment were taken (cell layer, predentin (proximal, central and distal), dentin and enamel). The corresponding area was calculated and the number of gold particles were counted and the result expressed as mean number of gold particles per µm^2^ (Au/µm^2^).

### Rat dental pulp mineralizing cell culture

rDPC were extracted and isolated from rat third molars according to the protocol by Lillesaar et al. [45]. The pulp was removed and placed in 10 mg collagenase with trypsin-EDTA solution for 2.5 hours at 37°C, with rotation. The suspension was drained through a cell strainer and centrifuged for 5 mins at 500 G. The cell pellet was resuspended in α-Minimal Essential Medium (α-MEM), 10% heat-inactivated fetal bovine serum (FBS) and 1% penicillin/streptomycin (P/S) (Invitrogen, Carlsbad CA, USA). Media was changed every other day.

A mineralizing phenotype was induced by seeding the rDPC at 28500 cells/cm^2^ in mineralizing media (α-MEM, 10% heat-inactivated FBS, 1% P/S, 50 µg/ml ascorbic acid phosphate and 10 mM β-glycerophosphate (Sigma)) [46]. Control experiments were run in parallel without the addition of osteogenic supplement. The experiments were run for 21 days and were repeated on three separate occasions.

### RNA extraction and quantitative real-time polymerase chain reaction (Q-RT-PCR)

Total RNA was isolated from cell cultures at d3, 7, 14 and 21 using the RNeasy MiniKit (Qiagen, Hilden Germany) according to the manufacturer's instructions. 1 µg of RNA was transcribed into cDNA using the High Capacity cDNA Reverse Transcription Kit (Applied Biosystems, Branchburg, New Jersey, USA).

Q-RT-PCR analyses were carried out using Applied Biosystems 7500 Fast Real-Time PCR system. The reactions were performed using 20 ng/ml cDNA, 5 µl TaqMan® Universal PCR Master Mix (Applied Biosystems), 7.5 µl RNase-free water and 1.5 µl specific primers; BGN (Assay ID: Rn00567229_m1*), DCN (Assay ID: Rn01503161_m1*), FMD (Assay ID: Rn00589918_m1*), OSAD (Assay ID: Rn00582664_m1*) OCN (Assay ID: Rn01455285_g1*) and rat GAPDH (endogenous control) (Assay ID: Rn01775763_g1*) (Applied Biosystems). The reactions were run at 50°C for 2 min, 95°C for 10 min followed by 40 cycles of 95°C 15 sec and 60°C for 1 min. All data were normalized with GAPDH gene expression values, and were analysed using the software DataAssist™ from Applied Biosystems, with the calculation of ddCT and normalised to GAPDH.

### Assessment of the mineralizing phenotype

Alizarin Red S staining, which detects calcium deposition, was used as an indicator of mineralization. Cells were rinsed in phosphate buffered saline (PBS, Invitrogen), and fixed in 70% ice-cold ethanol prior to staining with 40 mM Alizarin Red S (pH 4.2, Sigma) for 10 mins at RT. Calcium content was quantified by measu ring the amount of Alizarin Red S staining, which was bound to the mineralizing nodules. After staining, cultures were briefly washed PBS and extracted with 10% (w/v) cetylpyridinium chloride (Sigma) in 10 mM sodium phosphate pH 7.0 for 1 hr at RT. The concentration of the dye was determined by absorbance at 540 nm and was calculated based on the Alizarin red S standard used for staining.

### Statistics

All data are presented as means ± SEM and were compared using Students t-test (for 2 group's comparison). The value p<0.05 was considered as significant.

## Supporting Information

Figure S1 **Specificity of the mouse OSAD antibody was demonstrated by Western blotting.** A. Silver stain (Invitrogen) of 2.5 µg protein extracts and B. Western blot against mouse OSAD (1∶3000, R&D Systems) 0.5 µg of protein per lane. 1. d5 mouse incisor EDTA extraction (mineral-bound), 2. Adult incisor guanidine HCl extraction (non-mineral bound), 3. Adult incisor EDTA extraction (mineral-bound), 4 and recombinant mouse OSAD protein.
(DOC)

Figure S2 **Immunostaining of BGN.** At E15 (A) and 17 (B) BGN was not observed but by NB (C) BGN was detected with the initiation of dentinogenesis at the crown stage. Here the localization was proximal to the odontoblastic cell layer. This defined expression of BGN was continued throughout the remaining developmental stages (NB (C), d5 (D) and adult (E)) with no staining apparent in the alveolar bone, or enamel. Primary antibody was omitted in the control sections (Control) (F). A = ameloblasts, AB = alveolar bone, D = dentin, DF = dental follicle, E = enamel, pA = pre-ameloblasts, PD = predentin, pOB = pre-odontoblasts and OB = odontoblasts.
(DOC)

Figure S3 **Immunostaining for DCN.** DCN was not expressed during the early stages of tooth development at E15 (A) but was detected at the late bell stage with early dentinogenesis, and positive signal was also noted in the pulp complex (E17) (B). DCN was also localized to the alveolar bone surrounding the tooth (E17 to d5) (B–D) and in the predentin layer proximal to the odontoblastic cell layer throughout crown formation (NB to adult) (C–E). Control sections omitting the primary antibody showed no staining (F). A = ameloblasts, AB = alveolar bone, D = dentin, DF = dental follicle, E = enamel, pA = pre-ameloblasts, PD = predentin, pOB = pre-odontoblasts and OB = odontoblasts.
(DOC)

Figure S4 **Immunostaining for FMD.** During early development no immunostaining was apparent for FMD (E15-E17) (A, B), FMD was evident as dentinogenesis began in the crown stage in the NB (C) mouse incisors with some staining in the pulp complex and surrounding alveolar bone. FMD signal was noted in the predentin, proximal to the odontoblastic cell layer (NB to adult) (C–E). Control sections had the primary antibody omitted and showed no staining (F). AB = alveolar bone, A = ameloblasts, D = dentin, DF = dental follicle, E = enamel, pA = pre-ameloblasts, PD = predentin, pOB = pre-odontoblasts and OB = odontoblasts.
(DOC)

Figure S5 **Electron images of OSAD localization in the adult mouse molars.** OSAD expression in the different regions of the tooth were examined, predentin proximal (A), predentin distal (B), dentin (C) and enamel (D). Arrows indicate gold-labeled OSAD. An increased immunoreactivity in the highly active predentin (B) and to some extent in the dentin layer (C) were observed. Adsorption controls in NB, whereby the OSAD antibody was incubated with the recombinant protein showed no labeled gold particles (E).
(DOC)

Figure S6 **Quantification of gold-labeled OSAD particles in the predentin (proximal, central and distal), dentine and enamel following ultrastructural analysis of NB, d5 and adult molars.** The results are expressed as number of particles/µm^2^ (Au/µm^2^). Statistically significant differences (p<0.05) are denoted by *.
(DOC)
