# Supplementary material for: Osteoadherin Accumulates in the Predentin towards the Mineralization Front in the Developing Tooth
Source: PLoS One. 2012 Feb 15;7(2):e31525. doi: 10.1371/journal.pone.0031525 (PMC3280325; doi:10.1371/journal.pone.0031525)
Supplement: Figure S1 — Specificity of the mouse OSAD antibody was demonstrated by Western blotting. A. Silver stain (Invitrogen) of 2.5 µg protein extracts and B. Western blot against mouse OSAD (1∶3000, R&D Systems) 0.5 µg of protein per lane. 1. d5 mouse incisor EDTA extraction (mineral-bound), 2. Adult incisor guanidine HCl extraction (non-mineral bound), 3. Adult incisor EDTA extraction (mineral-bound), 4 and recombinant mouse OSAD protein. (DOC) [file pone.0031525.s001.doc]

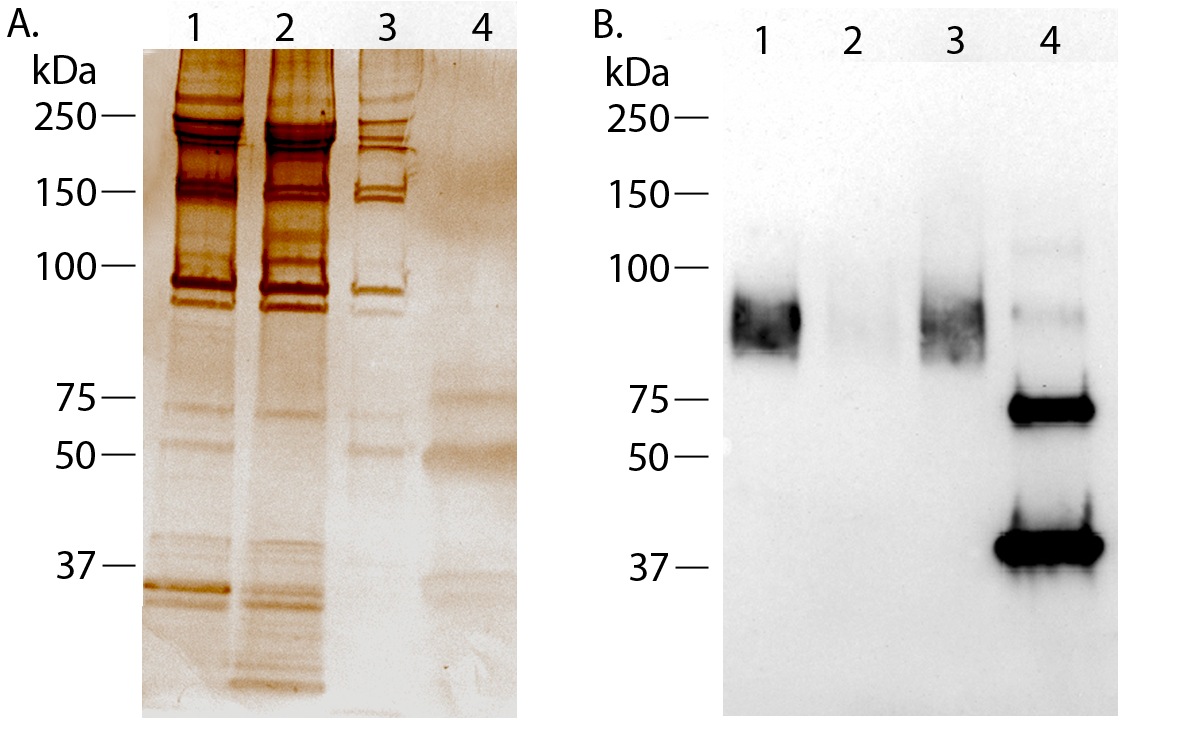


*Figure S1.*

Specificity of the mouse OSAD antibody was demonstrated by Western blotting. A. Silver stain (Invitrogen) of 2.5µg protein extracts and B. Western blot against mouse OSAD (1:3000, R&D Systems) 0.5µg of protein per lane. 1. d5 mouse incisor EDTA extraction (mineral-bound), 2. Adult incisor guanidine HCl extraction (non-mineral bound), 3. Adult incisor EDTA extraction (mineral-bound), 4 and recombinant mouse OSAD protein.
