# Supplementary material for: Osteoadherin Accumulates in the Predentin towards the Mineralization Front in the Developing Tooth
Source: PLoS One. 2012 Feb 15;7(2):e31525. doi: 10.1371/journal.pone.0031525 (PMC3280325; doi:10.1371/journal.pone.0031525)
Supplement: Figure S2 — Immunostaining of BGN. At E15 (A) and 17 (B) BGN was not observed but by NB (C) BGN was detected with the initiation of dentinogenesis at the crown stage. Here the localization was proximal to the odontoblastic cell layer. This defined expression of BGN was continued throughout the remaining developmental stages (NB (C), d5 (D) and adult (E)) with no staining apparent in the alveolar bone, or enamel. Primary antibody was omitted in the control sections (Control) (F). A = ameloblasts, AB = alveolar bone, D = dentin, DF = dental follicle, E = enamel, pA = pre-ameloblasts, PD = predentin, pOB = pre-odontoblasts and OB = odontoblasts. (DOC) [file pone.0031525.s002.doc]

*
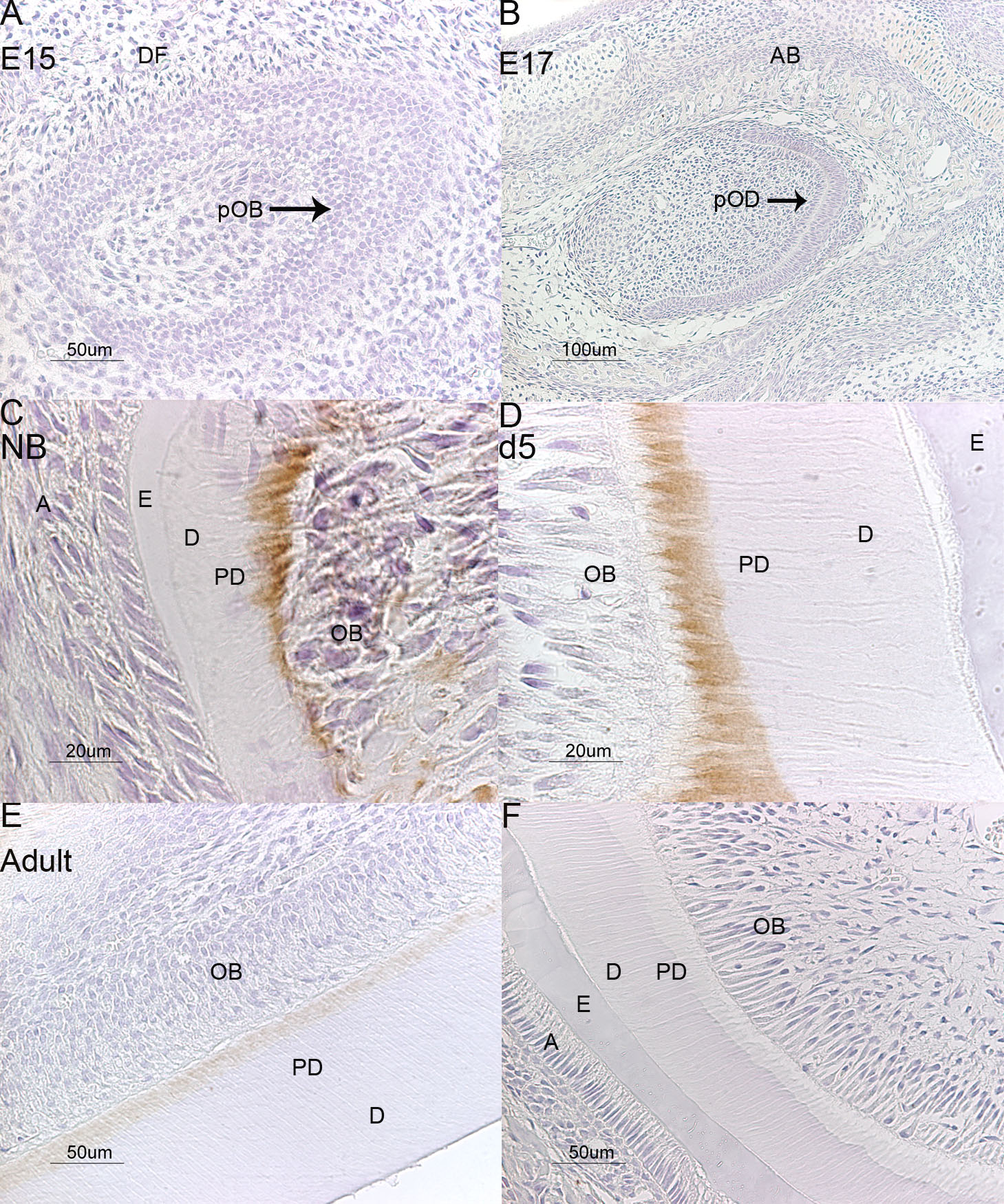
*

*Figure S2.*

Immunostaining of BGN. At E15 (A) and 17 (B) BGN was not observed but by NB (C) BGN was detected with the initiation of dentinogenesis at the crown stage. Here the localization was proximal to the odontoblastic cell layer. This defined expression of BGN was continued throughout the remaining developmental stages (NB (C), d5 (D) and adult (E)) with no staining apparent in the alveolar bone, or enamel. Primary antibody was omitted in the control sections (Control) (F).

*A = ameloblasts, AB = alveolar bone, D = dentin, DF = dental follicle, E = enamel, pA = pre-ameloblasts, PD = predentin, pOB = pre-odontoblasts and OB = odontoblasts*
