# Supplementary material for: Osteoadherin Accumulates in the Predentin towards the Mineralization Front in the Developing Tooth
Source: PLoS One. 2012 Feb 15;7(2):e31525. doi: 10.1371/journal.pone.0031525 (PMC3280325; doi:10.1371/journal.pone.0031525)
Supplement: Figure S3 — Immunostaining for DCN. DCN was not expressed during the early stages of tooth development at E15 (A) but was detected at the late bell stage with early dentinogenesis, and positive signal was also noted in the pulp complex (E17) (B). DCN was also localized to the alveolar bone surrounding the tooth (E17 to d5) (B–D) and in the predentin layer proximal to the odontoblastic cell layer throughout crown formation (NB to adult) (C–E). Control sections omitting the primary antibody showed no staining (F). A = ameloblasts, AB = alveolar bone, D = dentin, DF = dental follicle, E = enamel, pA = pre-ameloblasts, PD = predentin, pOB = pre-odontoblasts and OB = odontoblasts. (DOC) [file pone.0031525.s003.doc]

*
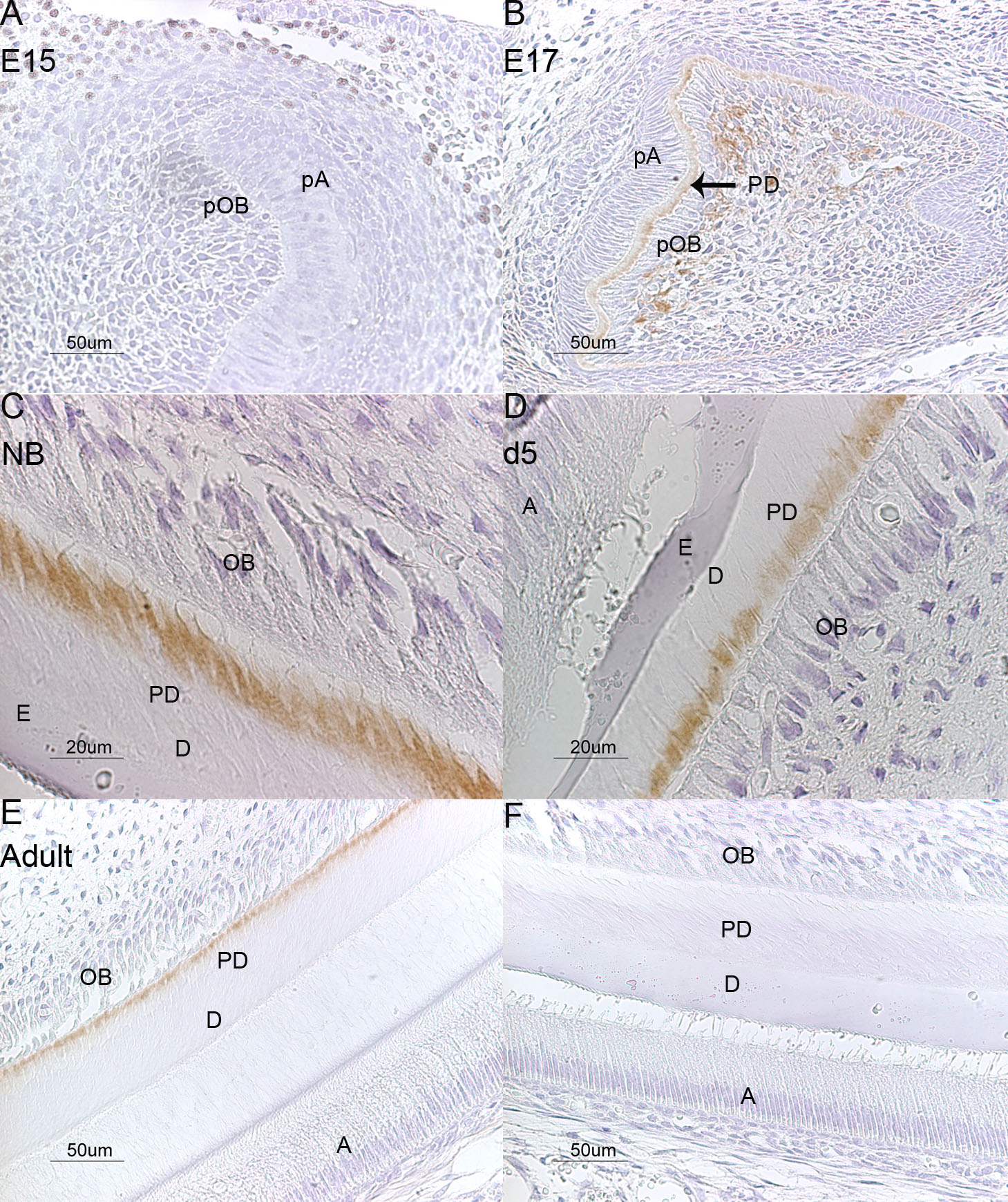
*

*Figure S3.*

Immunostaining for DCN. DCN was not expressed during the early stages of tooth development at E15 (A) but was detected at the late bell stage with early dentinogenesis, and positive signal was also noted in the pulp complex (E17) (B). DCN was also localized to the alveolar bone surrounding the tooth (E17 to d5) (B-D) and in the predentin layer proximal to the odontoblastic cell layer throughout crown formation (NB to adult) (C-E). Control sections omitting the primary antibody showed no staining (F).

*A = ameloblasts, AB = alveolar bone, D = dentin, DF = dental follicle, E = enamel, pA = pre-ameloblasts, PD = predentin, pOB = pre-odontoblasts and OB = odontoblasts*
