# Supplementary material for: Osteoadherin Accumulates in the Predentin towards the Mineralization Front in the Developing Tooth
Source: PLoS One. 2012 Feb 15;7(2):e31525. doi: 10.1371/journal.pone.0031525 (PMC3280325; doi:10.1371/journal.pone.0031525)
Supplement: Figure S4 — Immunostaining for FMD. During early development no immunostaining was apparent for FMD (E15-E17) (A, B), FMD was evident as dentinogenesis began in the crown stage in the NB (C) mouse incisors with some staining in the pulp complex and surrounding alveolar bone. FMD signal was noted in the predentin, proximal to the odontoblastic cell layer (NB to adult) (C–E). Control sections had the primary antibody omitted and showed no staining (F). AB = alveolar bone, A = ameloblasts, D = dentin, DF = dental follicle, E = enamel, pA = pre-ameloblasts, PD = predentin, pOB = pre-odontoblasts and OB = odontoblasts. (DOC) [file pone.0031525.s004.doc]

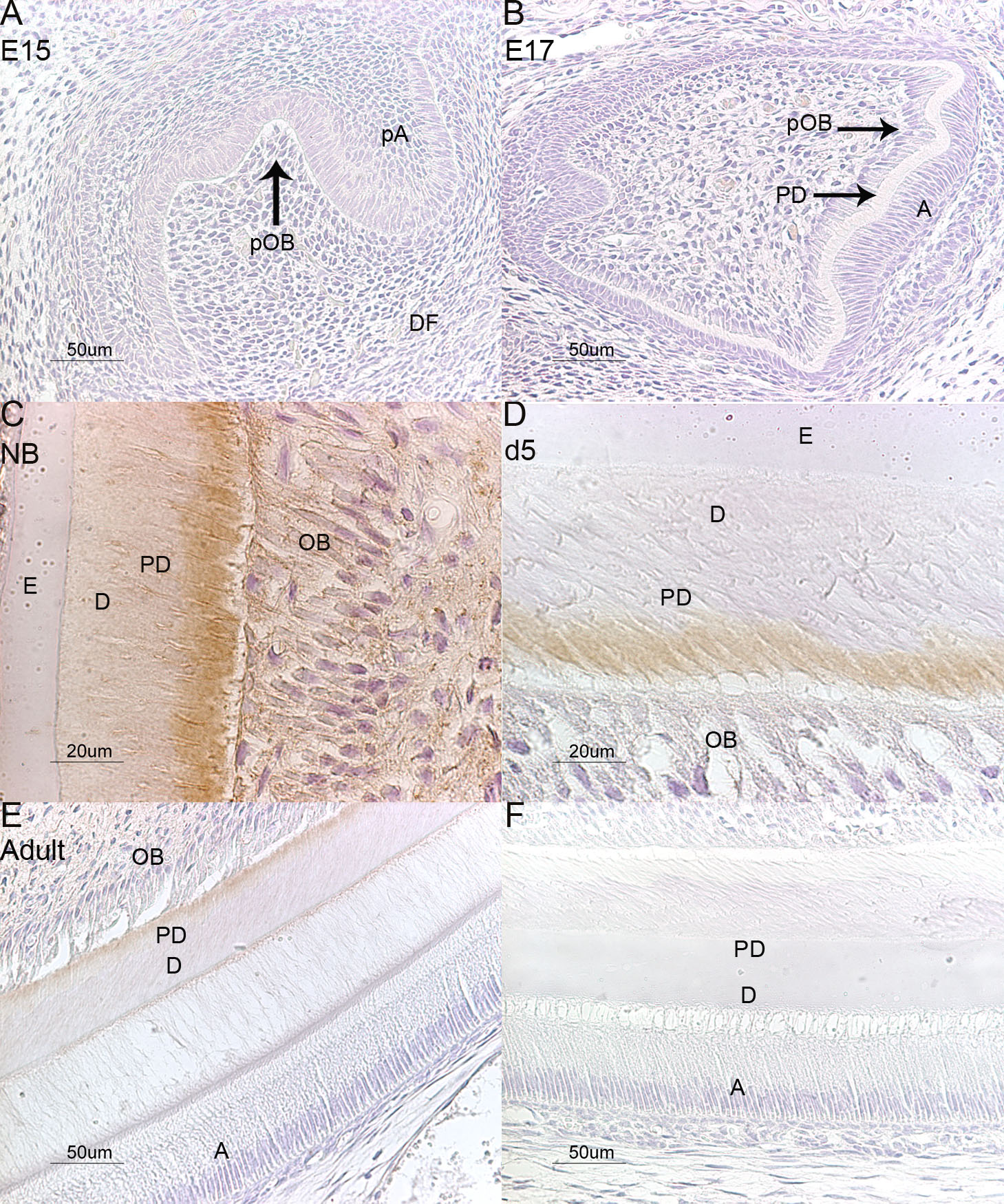


*Figure S4.*

Immunostaining for FMD. During early development no immunostaining was apparent for FMD (E15-E17) (A, B), FMD was evident as dentinogenesis began in the crown stage in the NB (C) mouse incisors with some staining in the pulp complex and surrounding alveolar bone. FMD signal was noted in the predentin, proximal to the odontoblastic cell layer (NB to adult) (C-E). Control sections had the primary antibody omitted and showed no staining (F).

*AB = alveolar bone, A = ameloblasts, D = dentin, DF = dental follicle, E = enamel, pA = pre-ameloblasts, PD = predentin, pOB = pre-odontoblasts and OB = odontoblasts*
