# Supplementary material for: Osteoadherin Accumulates in the Predentin towards the Mineralization Front in the Developing Tooth
Source: PLoS One. 2012 Feb 15;7(2):e31525. doi: 10.1371/journal.pone.0031525 (PMC3280325; doi:10.1371/journal.pone.0031525)
Supplement: Figure S5 — Electron images of OSAD localization in the adult mouse molars. OSAD expression in the different regions of the tooth were examined, predentin proximal (A), predentin distal (B), dentin (C) and enamel (D). Arrows indicate gold-labeled OSAD. An increased immunoreactivity in the highly active predentin (B) and to some extent in the dentin layer (C) were observed. Adsorption controls in NB, whereby the OSAD antibody was incubated with the recombinant protein showed no labeled gold particles (E). (DOC) [file pone.0031525.s005.doc]

*
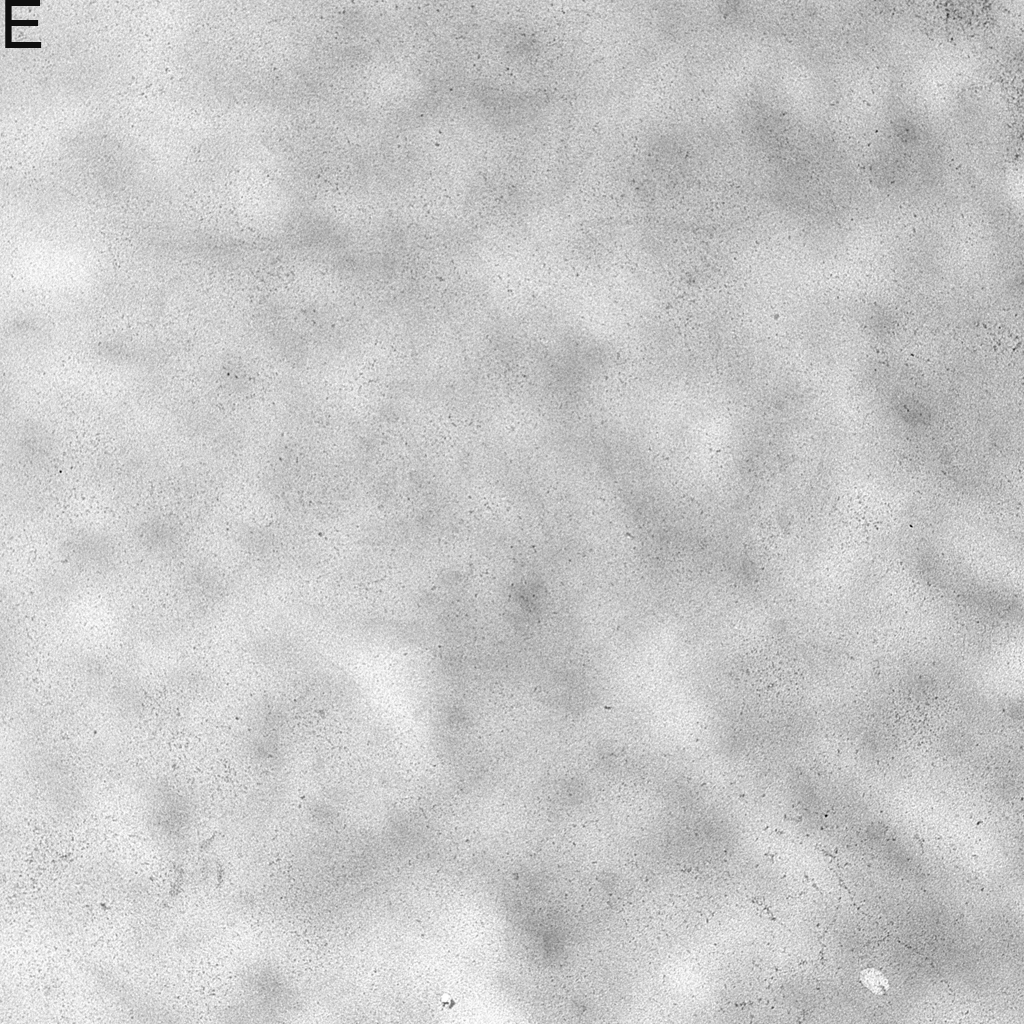

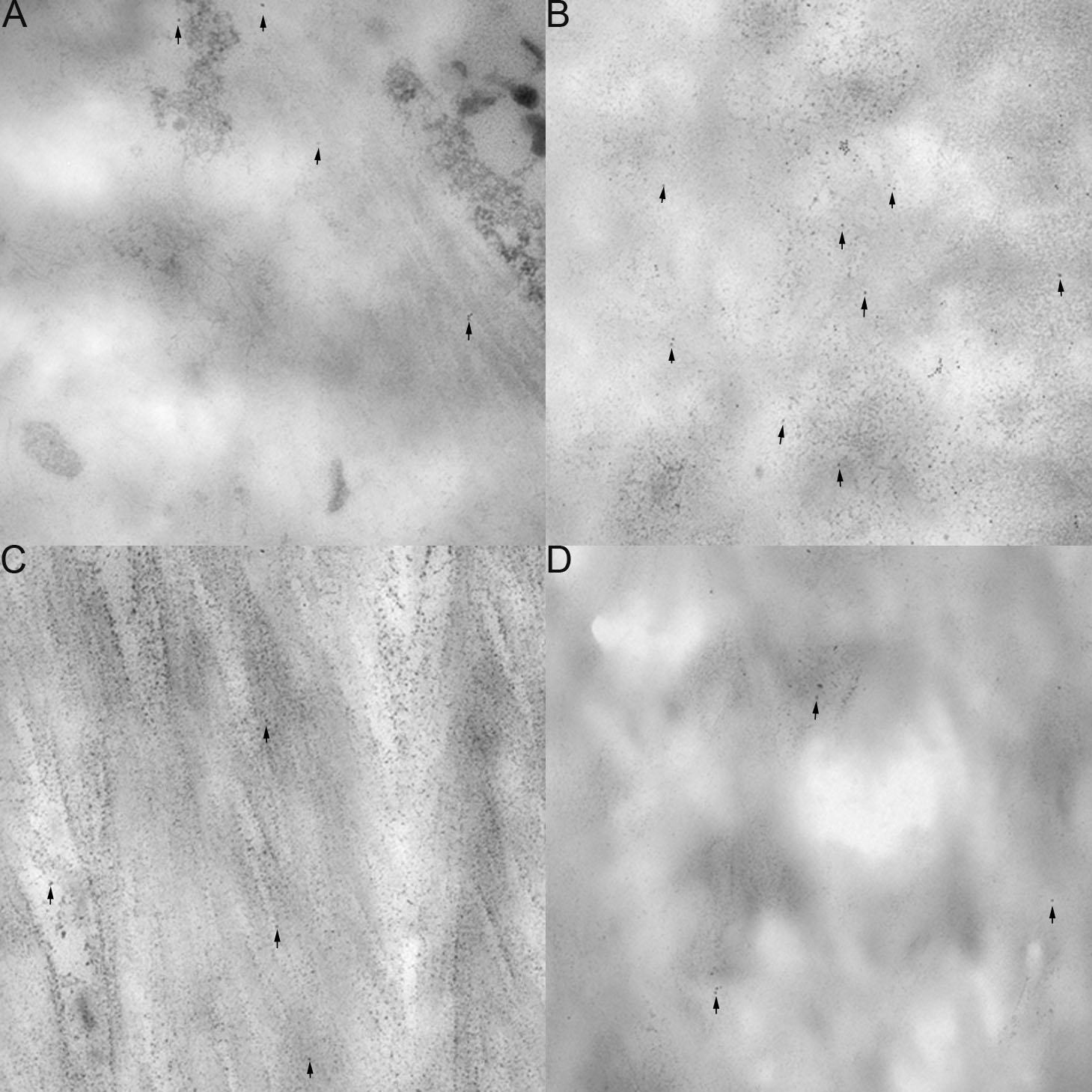
*

*Figure S5.*

Electron images of OSAD localization in the adult mouse molars. OSAD expression in the different regions of the tooth were examined, cell-layer (A), predentin (B), dentin (C) and enamel (D). Arrows indicate gold-labeled OSAD~~.~~ An increased immunoreactivity in the highly active predentin (B) and to some extent in the dentin layer (C) were observed. Throughout all developmental stages, few OSAD gold-labeled particles were detected in the cell-layer (A) an enamel (D). Adsorption controls in the NB, whereby the OSAD antibody was incubated with the recombinant protein showed no labeled gold particles (E).
