# Supplementary material for: Osteoadherin Accumulates in the Predentin towards the Mineralization Front in the Developing Tooth
Source: PLoS One. 2012 Feb 15;7(2):e31525. doi: 10.1371/journal.pone.0031525 (PMC3280325; doi:10.1371/journal.pone.0031525)
Supplement: Figure S6 — Quantification of gold-labeled OSAD particles in the predentin (proximal, central and distal), dentine and enamel following ultrastructural analysis of NB, d5 and adult molars. The results are expressed as number of particles/µm2 (Au/µm2). Statistically significant differences (p<0.05) are denoted by *. (DOC) [file pone.0031525.s006.doc]

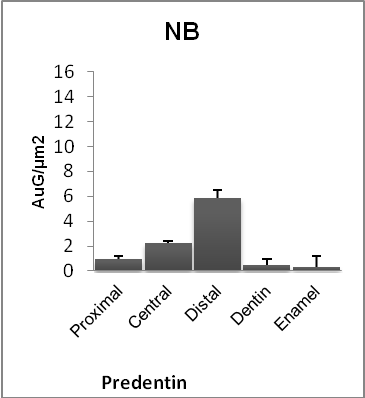

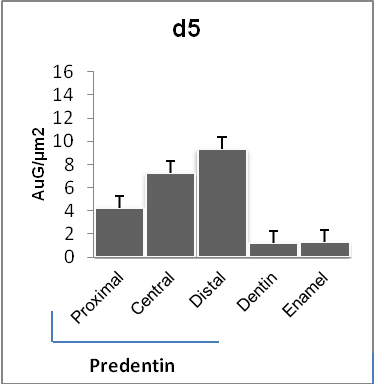

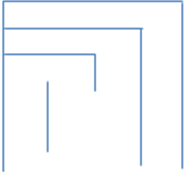

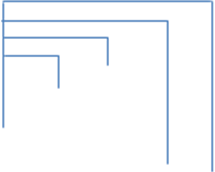


*

*

*

*

*

*

*

*


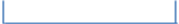


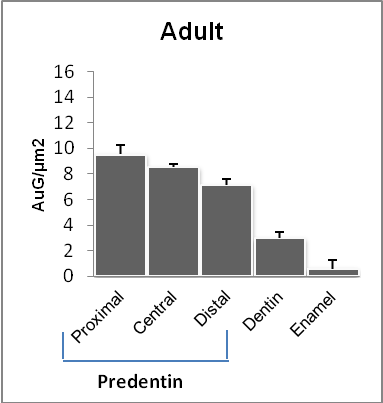


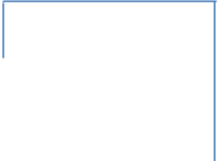


*

*Figure S6.*

Mean density of Au/µm^2^ (SEM) of OSAD in molars of developing mouse tooth; predentin (proximal, central, distal), dentin and enamel

| **Specimen Area** | **NB-a** | **d5-b** | **Adult-c** |
| --- | --- | --- | --- |
| **Predentin** |  |  |  |
| *1. Proximal* | 0.9 (0.3) | 4.3 (0.7) | 9.5 (0.7) |
| *2. Central* | 2.2 (0.2) | 7.3 (0.5) | 8.5 (0.2) |
| *3. Distal* | 5.8 (0.7) | 9.3 (0.8) | 7.1 (0.5) |
| **4. Dentine** | 0.5 (0.5) | 1.2 (0.8) | 2.1 (0.5) |
| **5. Enamel** | 0.3 (0.9) | 1.3 (0.4) | 0.6 (0.7) |


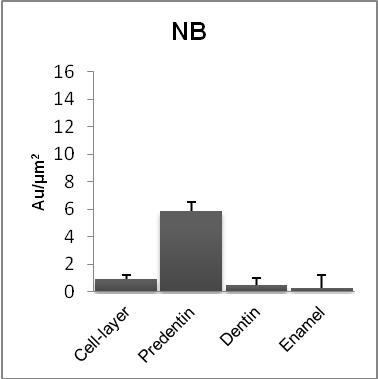


1vs 2, 1 vs 3, 1 vs 4 and 1 vs 5 were all compared for the different developmental stages, using Student’s *t* test. NS = statistically nonsignificant

.

**Molars (*P<0.05)**

a (NB): 1vs 2*, 1 vs 3* 1 vs 4* and 1 vs 5*
b (d5): 1vs 2*, 1 vs 3* 1 vs 4* and 1 vs 5*
c (Adult): 1vs 2 NS, 1 vs 3 NS 1 vs 4 NS and 1 vs 5*
